# Basing information on comprehensive, critically appraised, and up-to-date syntheses of the scientific evidence: a quality dimension of the International Patient Decision Aid Standards

**DOI:** 10.1186/1472-6947-13-S2-S5

**Published:** 2013-11-29

**Authors:** Victor M Montori, Annie LeBlanc, Angela Buchholz, Diana L Stilwell, Apostolos Tsapas

**Affiliations:** 1 Mayo Clinic, 200 First Street SW, Rochester, Minnesota, 55905, USA; 2 University Medical Center Hamburg-Eppendorf, Martinistraße 52, D - 20246 Hamburg, Germany; 3 Informed Medical Decisions Foundation, 40 Court Street, Suite 300, Boston, Massachusetts, 02108, USA; 4 Aristotle University, Thessaloniki, Konstantinupoleos 49, 54642, Greece

## Abstract

**Background:**

Patients and clinicians expect patient decision aids to be based on the best available research evidence. Since 2005, this expectation has translated into a quality dimension of the International Patient Decision Aid Standards.

**Methods:**

We reviewed the 2005 standards and the available literature on the evidence base of decision aids as well as searched for parallel activities in which evidence is brought to bear to inform clinical decisions. In conducting this work, we noted emerging and research issues that require attention and may inform this quality dimension in the future.

**Results:**

This dimension requires patient decision aids to be based on research evidence about the relevant options and the nature and likelihood of their effect on outcomes that matter to patients. The synthesis of evidence should be comprehensive and up-to-date, and the evidence itself subject to critical appraisal. Ethical (informed patient choice), quality-of-care (patient-centered care), and scientific (evidence-based medicine) arguments justify this requirement. Empirical evidence suggests that over two thirds of available decision aids are based on high-quality evidence syntheses. Emerging issues identified include the duties of developers regarding the conduct of systematic reviews, the impact of comparative effectiveness research, their link with guidelines based on the same evidence, and how to present the developers’ confidence in the estimates to the end-users. Systematic application of the GRADE system, common in contemporary practice guideline development, could enhance satisfaction of this dimension.

**Conclusions:**

While theoretical and practical issues remained to be addressed, high-quality patient decision aids should adhere to this dimension requiring they be based on comprehensive and up-to-date summaries of critically appraised evidence.

## Background

The original 2005 International Patient Decision Aid Standards (IPDAS) formulation included a key quality dimension then labeled as “basing information on up-to-date scientific evidence”. This dimension reflected the Standards panel’s belief that these tools should advance evidence-based practice. Since 2005, much work has taken place that advances the use of research evidence to develop tools that support clinical decision making, requiring a review and update in the formulation of this quality dimension. For this iteration, we put forward a more detailed description of this dimension. We now require patient decision aids to base their information on comprehensive, critically appraised, and up-to-date syntheses of the scientific evidence.

In this manuscript, we present a detailed description of this quality dimension, the theoretical and empirical justification for evaluating patient decision aids according to this dimension, and discuss the emerging issues and research areas for consideration and discussion identified during this endeavor.

## The dimension and its features

IPDAS requires patient decision aids to base their information on comprehensive, critically appraised, and up-to-date syntheses of the scientific evidence. This refers to the information about the various relevant options, and about the descriptions and likelihoods of those options’ effects on the outcomes of most importance to patients.

The term “scientific evidence” refers to a body of empirical observations about the options and their consequences. These observations should be made with some degree of protection against systematic and random error.

By “comprehensive”, we do not mean the scope and range of the information presented in the decision aid. Instead, in defining this quality dimension, “comprehensive” refers to the extent to which decision aid developers have thoroughly considered all the pertinent scientific evidence addressing each aspect that they chose to present in their decision aid.

The requirement that the evidence be “critically appraised” means that decision aid developers will use evidence derived from systematic reviews that: a) avoid selection bias (i.e., avoid introducing bias into the review in the selection of studies to include); b) carefully and reproducibly assess the quality of the incorporated reviewed studies (i.e., the studies’ protection from error and bias); c) summarize the estimated pertinent effects (ideally quantitatively in a meta-analysis); d) indicate the extent to which these estimates are trustworthy; and e) assess the extent to which selective reporting and publication bias may corrupt the body of evidence. Developers must present this synthesized evidence in the decision aid itself in a way such that it a) conveys that the aid is offering the “best available” synthesized information, and b) indicates—using symbols, numbers, or phrases—the degree of confidence attributed to that information, given the quality of the scientific evidence upon which it is based.

The dates when the relevant systematic reviews were searched for, compiled, critically appraised, and synthesized by decision aid developers should be reported and should be sufficiently recent given the pace of progress in the particular field to be considered “up-to-date”. This implies that decision aid developers should develop a sense of the speed with which evidence that matters accrues in their area of work, and implement an update policy. Developers should report a version date, and, when pertinent, a “better by” or “expiration date” to communicate to decision aid users about the speed of evidence accrual in the field and the optimal timing of future updates.

## Theoretical justification for evaluating patient decision aids on this quality dimension

As we described above, clinicians and patients expect patient decision aids to be evidence-based tools. This alone could justify this dimension (and indeed it did in its 2005 formulation). Here, we push the rationale further a) by connecting it with three fundamental arguments – an ethical argument, a quality-of-care argument, and a scientific argument – that are central to contemporary clinical care, and b) by describing recent advances in the rating of the degree of confidence in the estimates of effect justified by the state of the available scientific evidence.

### Supporting arguments

Three fundamental arguments central to contemporary clinical care justify the IPDAS’s evidence dimension.

#### Informed Patient Choice

One key principle driving the development of patient decision aids is the ethical argument for informing patients about their health care choices. Respect for patient autonomy is a governing principle of medical ethics [1], and is generally understood to refer to an individual’s ability to make and carry out informed health care decisions based on unbiased and thoughtful deliberation. The American Board of Internal Medicine, the American College of Physicians, and the European Federation of Internal Medicine state in their charter on medical professionalism that “physicians must be honest with their patients…and ensure that patients are completely and honestly informed before the patient has consented to treatment and after treatment has occurred.”[2] To be “completely and honestly informed” so that autonomy can be exercised requires access to unbiased information that is based on a high-quality synthesis of the available evidence that is relevant to the patient’s clinical situation and that acknowledges where uncertainty exists because of the quality or quantity of that evidence. When used as part of a shared decision making process, high-quality patient decision aids that are based on the best available clinical evidence support clinicians in fulfilling their ethical obligation to promote autonomy by ensuring that patients are fully informed about their health care choices.

#### Patient-Centered Care

The growing emphasis on patient-centered care is another driving force behind the development of patient decision aids. Principles of patient-centered care require that patients actively participate in decision making and be provided with the information and support they need to make informed choices. Work by the Picker Institute and others has identified respect for the patient’s values and preferences, as well as access to clear, *high-quality information* and education to be among the important characteristics of patient-centered care [3]. Patient decision aids support the practice of patient-centered care by ensuring that patients’ preferences are informed and based on accurate expectations; this requires, in turn, that the tools be based in high-quality evidence that, when possible, is relevant to patients’ individual risk profiles.

#### Evidence-Based Medicine

Over the last 20 years, evidence-based medicine (EBM) has strongly influenced the practice of medicine [4] as it has required use of the best available evidence alongside clinical expertise to formulate recommendations to patients that are pertinent to their context and sensitive to their values and preferences. To this extent, EBM follows two principles.

The first principle recognizes that not all observations and experiments are similarly protected from random and systematic error. The degree of protection from error offers confidence in the estimates of effect. Therefore, in the interests of fostering EBM, the information presented in patients’ decision aids should include not only evidence-based estimates of the effects of the various relevant options, but also an indication of the extent to which this evidence is protected from error (i.e., we can have confidence in the estimates of effect).

The second principle holds that the evidence alone is never sufficient to fully inform a clinical decision. The evidence will be considered during the transactions between the treating clinician with a particular level of expertise and the patient with unique goals, values, and preferences; furthermore, the evidence will be applied in a particular biological, psychological, and sociocultural context. Therefore, in the interests of fostering EBM, the information presented in patients’ decision aids needs to be directly applicable to the patients and practitioners using the decision aid, and tailored to individual patients’ characteristics.

Ethical (informed patient choice), quality (patient-centered care), and scientific (evidence-based medicine) justifications make it imperative that decision aids be based on comprehensive, critically appraised, and up-to-date scientific evidence.

### Rating the degree of confidence in the estimates of effect

Decision aid developers may want to include information in their tool that is based on different forms of evidence. Natural history and prognostic information, for instance, often requires the developer to use large and long observational studies. Adverse effects, particularly those that are rare, may be better characterized in case reports, and their linkage to exposures ascertained through case-control studies. This notion of a hierarchy of evidence has received much attention and has resulted in an emerging consensus in the practice guidelines movement about how to assess the confidence in the estimates of effect from the body of pertinent evidence: the GRADE approach. While we will now focus on this approach, we recognize that other approaches to grading the evidence exist, but they have substantial shortcomings that this method obviates. Furthermore, consistency in rating evidence from guideline to decision aid may facilitate the development of decision aids in conjunction with clinical practice guidelines efforts.

#### The GRADE Approach

GRADE (Grading of Recommendations Assessment, Development and Evaluation) is the most comprehensive approach developed for the purposes of formulating clinical practice guidelines[5]. It could be particularly helpful for decision aid developers, as an approach to explaining to patients the extent to which one can have confidence in the pertinent estimates of an option’s effects.

We will summarize the GRADE approach to grading evidence of effectiveness here, but developers should review the extensive published guidance (http://www.gradeworkinggroup.org).

A key feature of this approach is that the assessment of quality applies to the body of evidence (not just to the individual study). This includes: a) the likelihood of bias (from the absence of protective features such as concealed randomization, blinding of pertinent groups, and analysis of participants as randomized)[6]; b) the likelihood of reporting or publication bias [7]; c) inconsistency in results across studies [8]; d) any imprecision in the estimates of effect (e.g., wide confidence intervals)[9]; and d) the degree of indirectness, in which the results do not directly apply to the pertinent patients, the comparisons are inadequate, or the studies measure a surrogate of limited validity [10].

Randomized trials often provide high confidence in effect estimates. The task is to identify limitations that would reduce our confidence in those estimates. Observational studies often provide low confidence in effect estimates, limitations further reduce that confidence, but some features, such as strong evidence of a dose-response relationship or evidence of a very large effect [11], increase our confidence. Importantly, this confidence differs by outcome such that, for example, we may have more confidence in the effects of an intervention on patient important benefits while having low confidence in its effects on harms.

## Empirical evidence regarding this quality dimension

To evaluate the current practice of including evidence in decision aids, we examined the Ottawa Decision Aid Inventory (http://decisionaid.ohri.ca/azinvent.php). Out of 257 decision aids included in the inventory, 134 provided references to scientific evidence used, when they were last updated (n = 135), and whether they were available on the Internet (n = 134). In a random sample of those decision aids (n = 20), stratified to correct for differences between providers, ten aids used a high quality systematic review/meta-analysis (AMSTAR score [12] ranging from 2 to 11 on a scale of 1-11), and five aids were based on practice guidelines (AGREE II score [13] ranging from 1 to 6 on a scale of 1-7). Two decision aids used data from multiple sources of original research, whereas two used only a narrative review or an expert’s opinion or a single piece of original research as the sources for evidence. Four decision aids did not explicitly cite, hence we could not locate and evaluate, the evidence they used. One of the 20 aids explicitly stated an expiration date and an update policy, whereas eight refer to a policy statement of the complete contents on the provider website (as part of a site notice on the website, but not as part of the aid itself). Three used the GRADE system in their presentation to clarify the quality of the evidence to the user.

## Discussion

Linking the quality of a patient decision aid to the quality of the process by which developers comprehensively identified, synthesized, and appraised up-to-date evidence is a key dimension represented in the IPDAS. Furthermore, the little empirical evidence available suggests much room for improvement. The risk with standards is to suggest that no uncertainties remain or that innovation has stalled. Neither is true with relation to this topic. In working through these issues, the authors identified the following emerging topics and research areas for discussion. The process by which these areas were identified and are highlighted here does not pretend to be systematic or all-encompassing, but rather serves as a starting point for a community-wide conversation.

### How might developers decide what evidence used to inform a decision might be pertinent to the patients who are the intended audience of the tool?

The extent to which evidence is pertinent is subject to much judgment. Users may end up having low confidence in estimates of subgroup effects (e.g., subgroup effects that cannot be confirmed or are very imprecise), such that it is sometimes advisable to use estimates from the general population [14]. The challenge of applying evidence from somewhat different patients, interventions, or outcomes to the situation of interest falls under the general rubric of *indirectness *[10]. The degree with which these differences are likely to undermine the applicability of the evidence—that is, the extent of indirectness—reduces the confidence that the estimates of effect are correct, and this could be reflected in the decision aid. Efforts to improve the volume and quality of comparative effectiveness research [15] may enhance the evidence base for decision aids, because this research requires direct comparisons that matter to clinical stakeholders – that is, measuring the effect of interventions on outcomes of importance to patients.

### How often should systematic reviews be updated?

The frequency with which systematic reviews should be updated – and, by extension, the products derived from these reviews – is still subject to research. A comprehensive technical review commissioned by the Agency for Healthcare Research and Quality and published in 2007 [16] found that the median time for the emergence of a signal that a review should be updated (e.g., substantial new evidence of effectiveness or harm, new alternatives, revelations about the nature of the old evidence) was 5.5 years; yet about 25% of the reviews could benefit from updating within 2 years of publication. The authors were not able to identify predictors of more urgent review and suggested yearly surveillance of systematic reviews.

### How might we trust tailored estimates?

Decision aids often need to present absolute risk estimates that relate to the individual characteristics of the patient of interest. There is considerable uncertainty about such baseline risks [17]. Prognostic calculators offer the opportunity to tailor such risk estimates. Given the importance of these estimates, decision aid developers must report which calculator they are using and provide an assessment of its validity, a matter that often requires independent evaluation from the population from which the formula was derived, as well as comparisons with competing risk estimators [18].

### How might developers communicate their confidence in the estimates of effect?

Communicating the confidence that decision aid developers have in the estimates of the benefits and harms associated with the selected options would help decision aid users to make sense of the magnitude and trustworthiness of the estimates. The communication of this confidence in the evidence should be done in a way that is simple and understandable, yet remains precise, without adding cognitive burden on decision aid users.

There is limited empirical work on how to communicate confidence in effect estimates to stakeholders in general, let alone decision aid users. A systematic review of information in decision aids reported that very few decision aid developers had addressed or incorporated confidence in effect estimates in their tools [19]. The ways by which these developers were representing this concept varied greatly, from icons (i.e., bronze-gold medals), to verbal labels (i.e., high, moderate, low), and to numeric intervals, and these have yielded mixed results [19].

The GRADE approach for guidelines offers a simple way to report on confidence in effect estimates, using categorical labels—for example, “very low”, “low”, “moderate”, and “high” confidence. [20,21]. An obvious advantage gained from using the same approach in guidelines and in decision aids to rate confidence in effect estimates is that decision aid developers could use the evidence supporting current high-quality guidelines as their source for up-to-date evidence.

### Will linking information to evidence-based practice guidelines foster decision aid uptake?

A key issue with decision aids is their underuse in practice, despite increasing evidence of their effectiveness and surging policy support in some regions. Thus, efforts to explicitly link their design and content to clinical policy and workflow may facilitate their adoption. This supports in part our approach of linking the development and content of decision aids to state-of-the-art approaches to the development and content of evidence-based practice guidelines.

The “ideal” situation indicating the potential usefulness of a patient decision aid is one in which there is evidence producing high-confidence estimates linking options to outcomes, but the options are closely matched, and the choice of the “best” course of action will depend mostly on the patient’s values and preferences. Guideline developers following GRADE usually will offer a weak or conditional suggestion in those circumstances. A suggestion based on high-confidence evidence would require the incorporation of patient values and preferences for implementation, that is, would benefit from a decision aid. This linkage between guidelines and decision aids may affect the development and uptake of decision aids. Furthermore, implementation of the guidelines, for example, in quality improvement efforts, could then be linked to the implementation of decision aids for weak or conditional suggestions.

### What happens with patient decision aids that contradict extant practice guidelines?

A key aspect worthy of surveillance is the fate of decision aids that have been developed based on the best available evidence, but which disagree in their presentation with extant guidelines and with the quality-of-care parameters derived from these guidelines. These discrepancies can appear because decision aids may use more recently updated summaries of evidence, or because the recommendations apply to a different context or resulted from panels with distorted values (e.g., due to pharmaceutical lobbying). In one example, this apparent divergence led to difficulties in the use of the decision aid and to its nonuse [22].

### Will adherence to this dimension improve decision aid use in practice?

Given that the uptake in practice of decision aids remains limited, there is no strong evidence that decision aids supported by high quality and updated summaries of the body of evidence would be more likely to be taken up in practice. It is plausible, however, that adherence to this domain may create a reputation for high credibility that could drive the uptake of decision aids. There is no *a priori* reason to believe that the quality of the process of evidence synthesis and its adaptation into evidence-based decision aids will lead to better decision processes and outcomes. Yet, basing their content on accurate rather than inaccurate estimates may lead to better outcomes. These central concerns, therefore, remain important research questions.

## Conclusions

A key quality dimension for patient decision aids is how developers identified, summarized, and used research evidence to inform the content of these tools. Patients and clinicians have the expectation that these tools will support evidence-based practice. Ethical, quality-of-care, and scientific arguments justify making this an IPDAS standard. Empirical evidence suggests that there is much room for improvement in developers’ adherence to this standard. Applying the methodological developments of the GRADE working group to this task may help developers enhance their adherence to this domain and thus the quality of their patient decision aids. It may also help position the development of decision aids in the same development stream as that of high quality clinical practice guidelines. Yet, much room exists for research and innovation into the processes that create patient decision aids based on comprehensive and up-to-date syntheses of all the pertinent and critically appraised scientific evidence.

## List of abbreviations used

AGREE II: Appraisal of Guidelines for Research and Evaluation, second version; AMSTAR: Assessment of Multiple Systematic Reviews; EBM: Evidence-based Medicine; GRADE: Grading of Recommendations Assessment, Development and Evaluation; IPDAS: International Patient Decision Aid Standards.

## Competing interests

VMM is a member of the IPDAS Steering Committee and of the GRADE Working Group. His research includes the development of patient decision aids. He does not receive financial compensation for any of these activities, and his research group, the KER UNIT, does not receive funding from for-profit pharmaceutical or device manufacturers. Furthermore, the decision aids developed by the KER UNIT are freely available (http://shareddecisions.mayoclinic.org).

AL, also a researcher with the KER UNIT, investigates the development, evaluation, and implementation of decision aids at the point of care, and does not receive financial compensation for any of these activities.

AB’s research group on substance abuse treatment does not have any connection with pharmaceutical industry that might be related to the current research, research contracts, consultancy, employment or stocks.

DS receives salary support as Chief Production Officer for the Informed Medical Decisions Foundation, a not-for-profit (501 (c)3) private foundation (http://www.informedmedicaldecisions.org). The Foundation develops content for patient education programs. The Foundation has an arrangement with a for-profit company, Health Dialog, to co-produce these programs. The programs are used as part of the decision support and disease management services Health Dialog provides to consumers through health care organizations and employers.

AΤ’s research includes the implementation of decision aids. He does not have any connection with the pharmaceutical industry that might be related to the current research.

## Authors’ contributions

VMM created the first draft of this manuscript and contributed to discussions and critical revisions of it.

AL contributed to the writing, discussion, and critical revision of the manuscript.

AB contributed to the search and evaluation the current practice of reporting evidence in decision aids (section D) as well as discussion and critical revision of the whole manuscript.

DS contributed to discussions and critical revisions of the whole manuscript.

AT contributed to the search and appraisal of evidence reporting in decision aids, as well as discussion and critical revision of the whole manuscript.

## Authors’ information

VMM is a clinician and Professor of Medicine at Mayo Clinic in the United States, and has over a decade of experience developing and evaluating decision aids for patients with chronic conditions, conducting systematic reviews of healthcare interventions and formulating practice guidelines, and conducting research about evidence-based medicine, patient-centered care, and clinical practice guidelines. He leads the Shared Decision Making National Resource Center at Mayo Clinic.

AL is Assistant Professor of Health Services Research at Mayo Clinic, Rochester, MN, USA. She works in the development, evaluation, and implementation of shared decision making interventions, including decision aids, in both acute and chronic care settings.

AB is Senior Researcher at the Department of Medical Psychology at the Hamburg University Medical Center and has been involved in the development and evaluation of several shared decision-making interventions in various health contexts for the past four years.

DS is Chief Production Officer at the Informed Medical Decisions Foundation, Boston, Massachusetts, USA. Over the past decade, she has been involved in the development, evaluation, and implementation of dozens of decision aids.

AT is Assistant Professor of Medicine at Aristotle University, Thessaloniki, Greece, with a special interest in evidence-based medicine, evidence synthesis, and use of decision aids for chronic conditions.

## Declarations

Publication of this supplement was funded by an unrestricted grant from the Informed Medical Decisions Foundation and by the Center for Science of Healthcare Delivery, Mayo Clinic (Montori). Administrative and editorial support for this supplement was provided by The University of Texas MD Anderson Cancer Center.

This article has been published as part of *BMC Medical Informatics and Decision Making* Volume 13 Supplement 2, 2013: The International Patient Decision Aid Standards (IPDAS) Collaboration’s Quality Dimensions: Theoretical Rationales, Current Evidence, and Emerging Issues. The full contents of the supplement are available online at http://www.biomedcentral.com/bmcmedinformdecismak/supplements/13/S2.
